# Intratumoral *Enterobacter hormaechei* drives gemcitabine resistance in pancreatic cancer via *cdd*_L_‐mediated drug inactivation

**DOI:** 10.1002/imt2.70126

**Published:** 2026-04-29

**Authors:** Jun‐Feng Peng, Meixia Li, Ting Niu, Judong Li, Wei Niu, Minghui Zheng, Quanjiang Ji, Chuan Li, Chenghao Shao, Kan Ding

**Affiliations:** ^1^ Carbohydrate‐Based Drug Research Center, CAS Key Laboratory of Receptor Research, State Key Laboratory of Drug Research, Shanghai Institute of Materia Medica, Chinese Academy of Sciences Shanghai China; ^2^ Department of Pancreatic‐Biliary Surgery Second Affiliated Hospital of Naval Medical University Shanghai China; ^3^ State Key Laboratory of Drug Research, Shanghai Institute of Materia Medica, Chinese Academy of Sciences Shanghai China; ^4^ School of Physical Science and Technology & State Key Laboratory of Advanced Medical Materials and Devices Shanghai Tech University Shanghai China; ^5^ Zhongshan Institute for Drug Discovery, Shanghai Institute of Materia Medica Chinese Academy of Sciences, SSIP Healthcare and Medicine Demonstration Zone Zhongshan China

## Abstract

Gemcitabine resistance poses a critical barrier to improving survival in pancreatic cancer, yet the microbial drivers remain elusive. By integrating 16S rRNA amplicon sequencing with large‐scale culturomics across 114 clinical samples, we identified *Enterobacter hormaechei* as a key intratumoral pathogen. We demonstrate that *E. hormaechei* confers resistance by enzymatically converting the drug to its inactive metabolite dFdU via a unique long‐isoform cytidine deaminase encoded by *cdd*_L_. Kinetic analysis revealed exceptional catalytic efficiency (*K*_m_ = 0.22 mM, *k*_cat_ = 194.05 s^−1^), and genetic ablation of *cdd*_L_ fully restored drug sensitivity. In vivo, antibiotic co‐treatment eliminated intratumoral bacteria and potentiated gemcitabine efficacy, enabling a 50% dosage reduction without comprising therapeutic outcome. Pan‐cancer analysis further confirmed the broad prevalence of *Enterobacter* across multiple solid tumor types. These findings elucidate a *cdd*_L_‐mediated mechanism of chemoresistance and identify intratumoral *E. hormaechei* as a tractable therapeutic target for optimizing gemcitabine‐based regimens and improving patient outcomes.

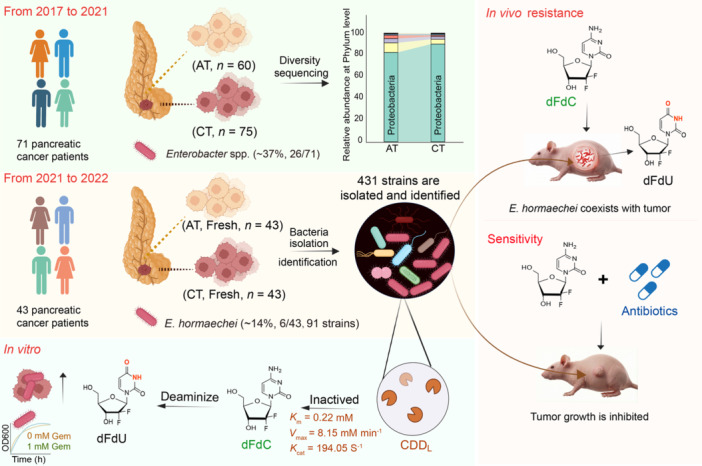

## CONFLICT OF INTEREST STATEMENT

The authors declare no conflicts of interest.

## ETHICS STATEMENT

All patients provided written informed consent prior to sample collection and the use of their clinical data for research purposes. All the clinical pancreatic cancer tissue collections were approved by the Second Affiliated Hospital of Naval Medical University Biomedical Research Ethics Committee with an approval number 2018SL004. All animal (SPF mice) experiments were performed following the current guidelines of the protocol IACUC (2021‐05‐DK‐99, 2022‐08‐DK‐109, 2023‐05‐DK‐119, 2024‐07‐DK‐138) approved by the Institutional Animal Care and Use Committee of Shanghai Institute of Materia Medica, following the Shanghai Institute of Materia Medica of Health Guide for the Care and Use of Laboratory Animals.

To the Editor,

Pancreatic ductal adenocarcinoma (PDAC) remains one of the most lethal malignancies, with a 5‐year survival rate of merely 13% [1]. Gemcitabine serves as the cornerstone of systemic therapy; however, its clinical benefit is substantially undermined by intrinsic and acquired drug resistance [2, 3], the mechanism basis of which remains incompletely defined. Emerging evidence indicates that the tumor microenvironment is not sterile [4, 5, 6, 7, 8]; specifically, intratumoral bacteria can metabolize chemotherapeutic agents, thereby limiting their cytotoxic potential [9, 10]. Notably, certain Gammaproteobacteria express the long‐isoform cytidine deaminase (CDD_L_) that catalyzes the deamination of gemcitabine to the inactive 2',2'‐difluorodeoxyuridine (dFdU) [10]. Nevertheless, previous mechanistic studies have predominantly relied on laboratory model strains, and the specific clinical isolates responsible for driving resistance within the PDAC microenvironment remain poorly characterized. *Enterobacter hormaechei*, a member of the *Enterobacter cloacae* complex, is a recognized opportunistic pathogen [11, 12, 13]; however, its colonization of tumor tissue and putative contribution to chemoresistance have not previously been reported. To address this gap, we employed comprehensive culturomics to isolate and characterize viable intratumoral bacteria from PDAC patients. Here, we identify *E. hormaechei* as one of the principal drivers of gemcitabine resistance, elucidate the underlying enzymatic mechanism mediated by bacterial *cdd*_L_ and validate a combinatorial antibiotic strategy in vivo. These findings establish the intratumoral *Enterobacter*‐gemcitabine axis as a novel therapeutic target and provide a conceptual framework for precision microbiota‐modulated oncology.

### PROFILING AND ISOLATION OF INTRATUMORAL *ENTEROBACTER HORMAECHEI*

**FIGURE 1:**
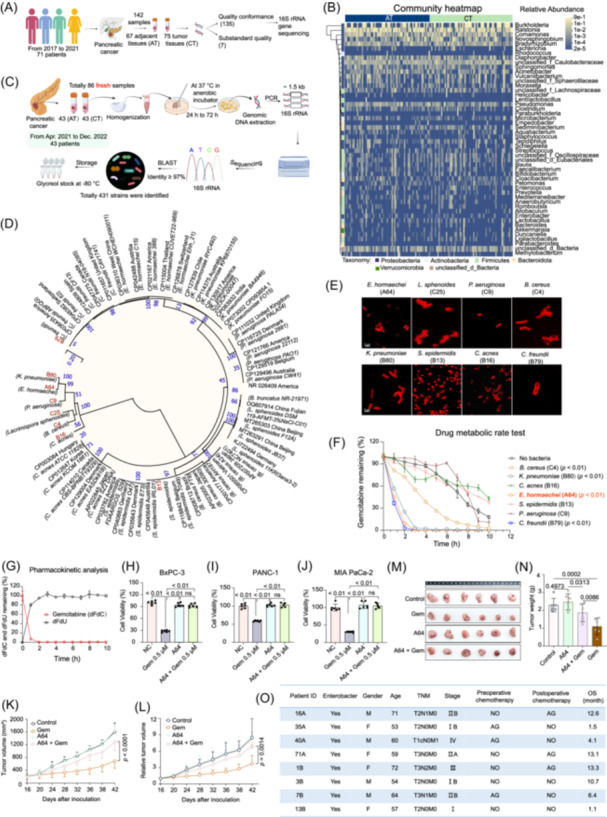
Identification and functional characterization of intratumoral *Enterobacter hormaechei* as a driver of gemcitabine resistance in pancreatic ductal adenocarcinoma (PDAC). (A) Schematic overview of the 16S rRNA gene sequencing discovery cohort (2017–2021), comprising 71 PDAC patients with adjacent tissues (AT, *n* = 67) and cancer tissues (CT, *n* = 75). (B) Heatmap illustrating the relative abundance of differentially represented microbial taxa between AT and CT samples, as determined by 16S rRNA sequencing. (C) Schematic overview of the independent culturomics validation cohort (2021–2022), comprising 43 patients. (D) Phylogenetic tree of intratumoral bacteria isolates reconstructed using MEGA11.0. (E) Fluorescence microscopy image of representative intratumoral bacterial isolates labeled with fluorescent d‐amino acids, including *E. hormaechei* strain A64. (F) Screening of bacterial isolates for gemcitabine‐metabolizing capacity. Strain A64 (orange) exhibited the most rapid drug degradation. (G) Pharmacokinetic time‐course analysis of strain A64 demonstrating complete conversion of gemcitabine (dFdC) to its inactive deamination product dFdU. In vitro cell viability assays (*n* = 6) in BxPC‐3 (H), PANC‐1 (I), and MIA PaCa‐2 (J) PDAC cell lines co‐cultured with or without strain A64 under gemcitabine treatment. (K–N) In vivo therapeutic efficacy evaluation in a subcutaneous xenograft mouse model (*n* = 6). (K) Tumor volume growth curves and (L) relative tumor volume changes over the treatment period. (M) Representative photographs of excised tumors at study endpoint. (N) Tumor weights at study endpoint. Intratumoral colonization by A64 significantly compromised gemcitabine therapeutic efficacy relative to the Gemcitabine‐only control group. (O) Clinical characteristics and outcomes summary for *Enterobacter*‐positive patients, illustrating the association between intratumoral colonization and adverse prognosis. All data are presented as the mean ± SD. Statistical analyses: (F) two‐way ANOVA with Dunnett's multiple comparisons test; (H)–(J) and (N) one‐way ANOVA with Tukey's multiple comparisons test; (K) and (L) two‐way repeated‐measures ANOVA with Tukey's post hoc test.

To systematically characterize the PDAC microbiome, we performed 16S rRNA gene sequencing on 135 clinical samples from a discovery cohort of 71 patients (Figure 1A). Taxonomic profiling revealed that Proteobacteria dominated the landscape, primarily driven by Betaproteobacteria, Alphaproteobacteria, and Gammaproteobacteria (Figure S1A,B). While the overall community diversity was similar between tumor and adjacent tissues (Figure S2A–E), detailed genus‐level analysis identified Enterobacter spp. in 37% (26/71) of patients (Figure 1B, Tables S1,S2), a prevalence further corroborated by a public database (Table S3).

To validate bacterial viability, we subsequently conducted large‐scale culturomics on fresh paired tissues from an independent validation cohort of 43 patients (Figure 1C, Table S4). This approach yielded 431 viable strains, confirming a metabolically active intratumoral ecosystem (Tables S5, S6). *Enterobacter hormaechei* was the most abundant species recovered (91 strains), followed by *Escherichia fergusonii* and *Cutibacterium acnes* (Table S7). Notably, *E. hormaechei* was successfully cultured from ~14% (6/43) of tumors. Clinical follow‐up revealed a striking association with adverse prognosis: five of the six patients harboring *Enterobacter hormaechei* died within 1 year of diagnosis. Phylogenetic analysis of eight representative isolates clustered *E. hormaechei* within the *Gammaproteobacteria* (Table S8, Figure 1D), and in situ bacterial presence was confirmed by fluorescent d‐amino acid labeling and immunohistochemistry (IHC) (Figure 1E, Figure S3A–H). Collectively, these multi‐modal data establish *E. hormaechei* as a predominant, viable, and clinically significant constituent of the PDAC tumor microenvironment.

### INTRATUMORAL *ENTEROBACTER HORMAECHEI* DRIVES GEMCITABINE RESISTANCE

To assess the functional consequence of intratumoral bacteria colonization, we systemically screened clinical isolates for gemcitabine‐inactivating capacity. Although drug metabolism was phylogenetically distributed, extending to members of Bacillota (e.g., *Lacrimispora sphenoides*), *E. hormaechei* (A64) exhibited the most potent inactivating activity (Figure S4A,B). Biochemical characterization confirmed that strain A64 is closely related to the type strain DSM101093 (Table S9). Pharmacokinetic analysis demonstrated that A64 rapidly and completely converted gemcitabine (dFdC) into its inactive metabolite dFdU within 2 h (Figure 1F,G). This enzymatic inactivation had profound biological consequences: A64 colonization significantly abrogated gemcitabine cytotoxicity across multiple PDAC cell lines in vitro (Figure 1H–J, Figure S5), and intratumoral A64 colonization severely compromised gemcitabine efficacy in a xenograft mouse model, yielding significantly greater tumor burden compared to bacteria‐free controls (Figure S6, Figure 1K–N). Consistent with these pre‐clinical observations, analysis of clinical outcome data revealed a counterintuitive finding: early‐stage patients (Stage IB) who received preoperative chemotherapy exhibited significantly shorter survival (1.5 months) compared to those with metastatic disease or those treated post‐operatively (Figure 1O). Comprehensive clinicopathological correlation analysis of the full cohort (*n* = 71) identified no significant correlations between *Enterobacter* colonization and TNM stage or tumor differentiation (*p* > 0.05), suggesting that this microbial resistance mechanism is an independent risk factor ubiquitous across the patient population (Table S10). Collectively, these findings establish *E. hormaechei* as a clinically relevant and potential driver of gemcitabine resistance in PDAC.

### CYTIDINE DEAMINASE DRIVES GEMCITABINE RESISTANCE IN *ENTEROBACTER HORMAECHEI*

**FIGURE 2:**
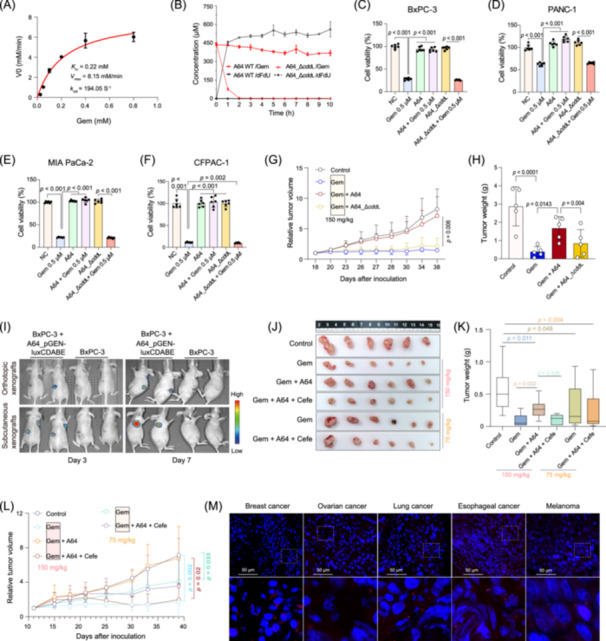
The long‐isoform cytidine deaminase (CDD_L_) is the mechanistic driver of *Enterobacter hormaechei*‐mediated gemcitabine resistance and a candidate therapeutic target. (A) Michaelis–Menten kinetic analysis of the purified recombinant CDD_L_ protein, showing substrate affinity and catalytic parameters for gemcitabine deamination. (B) Time‐dependent metabolic assay quantifying gemcitabine (Gem) depletion and concomitant dFdU accumulation in bacterial culture supernatants from wild‐type A64 and the isogenic *cdd*_L_‐deficient mutant (A64_Δ*cdd*_L_) (*n* = 3). In vitro cell viability assays (*n* = 6) in BxPC‐3 (C), PANC‐1 (D), MIA PaCa‐2 (E), and CFPAC‐1 (F) cell lines, comparing the gemcitabine‐resistance‐conferring capacity of wild‐type A64 versus A64_Δ*cdd*_L_. (G, H) In vivo validation of the *cdd*_L_‐dependent resistance mechanism in a subcutaneous xenograft mouse model (*n* = 5). (G) Relative tumor volume growth curves and (H) final tumor weights at study endpoint. (I) In vivo bioluminescence imaging of mice bearing orthotopic or subcutaneous xenograft tumors, demonstrating stable intratumoral colonization and long‐term persistence of *E. hormaechei*. (J–L) Assessment of antibiotic‐based therapeutic intervention and gemcitabine dosage optimization (*n* = 6). (J) Representative photographs of excised tumors from each treatment group. (K) Tumor growth curves and (L) final tumor weights at study endpoint. (M) Detection of intratumoral bacteria across diverse solid tumor types by 16S rRNA fluorescence in situ hybridization (*n* = 5). All data are presented as mean ± SD. Statistical analyses: (C)–(F), (H), and (L) one‐way ANOVA with Tukey's multiple comparisons test. (G) and (K) Two‐way repeated‐measures ANOVA with Tukey's post hoc test.

Given the established role of CDD_L_ in gemcitabine inactivation across both prokaryotes and eukaryotes [10, 14, 15, 16], we performed whole‐genome sequencing on *E. hormaechei* isolate A64 and identified a unique long‐isoform cytidine deaminase gene (*cdd*_L_) sharing 77.5% nucleotide identity with its *E. coli* counterpart (Figure S7A,B, Figure S8). Bioinformatic analysis confirmed the presence of this *cdd*_L_ gene in six additional species recovered from our isolate library (Table S11). Kinetic characterization of purified CDD_L_ demonstrated efficient gemcitabine deamination (*K*_m_ = 0.22 mM, *k*_cat_ = 194.05 s^−1^) (Figure S9A, Figure 2A). Notably, this catalytic efficiency markedly outperforms the CDDs homolog from *Enterococcus faecium*, which exhibits a significantly lower affinity (*K*_m_ = 8.44 mM) [17]. To establish causality, we constructed a *cdd*_L_‐deficient mutant (A64_Δ*cdd*_L_), which was entirely unable to metabolize gemcitabine (Figure S9B–F, Figure 2B). In pancreatic cancer cell line co‐culture assays and in xenograft models, A64_Δ*cdd*_L_ failed to confer drug resistance, and gemcitabine efficacy was fully restored to levels indistinguishable from drug‐only controls (Figure 2C–H, Figures S10A–D, S11A–C). These results identify CDD_L_‐mediated enzymatic inactivation as the primary mechanism underlying *Enterobacter*‐driven chemoresistance.

### ANTIBIOTIC‐MEDIATED ERADICATION RESTORES GEMCITABINE EFFICACY AND IMPLICATES *ENTEROBACTER* AS A PAN‐CANCER THERAPEUTIC TARGET

Using a bioluminescent *E. hormaechei* strain (Figure S12A–C), we tracked bacteria dynamics within the tumor microenvironment in real time, confirming rapid proliferation and sustained intratumoral persistence (Figure 2I, Figure S12D–F). These observations suggest a temporally and dynamic role for bacterial colonization: while initial tumor seeding may be an incidental consequence of altered tissue physiology during tumorigenesis, the ensuring metabolic activity of resident bacteria constitute an acquired, functionally significant driver of chemotherapy failure. To validate a therapeutic strategy targeting this mechanism, we assessed cefepime, a broad‐spectrum cephalosporin. Cefepime showed no intrinsic antitumor activity (Table S12, Figure S13A–C); however, its co‐administration significantly reduced intratumoral bacterial burden and restored gemcitabine sensitivity in vivo (Figure 2J–L, Figure S14A–C). Remarkably, the combination of cefepime with low‐dose gemcitabine (75 mg/kg) achieved antitumor efficacy comparable to the high‐dose regimen (150 mg/kg) (Figure 2J–L), suggesting a potential strategy for dosage optimization. However, we believe the ultimate clinical solution lies not in long‐term antibiotic use, but in the development of small‐molecule inhibitors targeting the CDD_L_ enzyme. Finally, extending the translational scope of these findings, IHC, FISH, and database mining revealed a substantial *Enterobacter* spp. prevalence across breast, lung, melanoma, esophageal and particularly ovarian cancers (Figure 2M, Figure S15A–F, Table S13). These results suggest that *Enterobacter*‐mediated drug inactivation is not unique to pancreatic cancer but may represent a ubiquitous mechanism of chemoresistance in diverse solid malignancies.

This study demonstrates that intratumoral *E. hormaechei*, prevalent across a spectrum of solid tumors, drives chemoresistance through highly efficient CDD_L_‐mediated inactivation of gemcitabine. Targeted bacteria eradication with antibiotics not only fully reverses this resistance but also enables a 50% reduction in the effective chemotherapy dose, offering a highly clinically translatable paradigm to overcome microbe‐driven treatment failure.

## AUTHOR CONTRIBUTIONS

**Jun‐Feng Peng**: Investigation; methodology; data curation. **Meixia Li**: Conceptualization; investigation; supervision; project administration; writing—original draft; funding acquisition; writing—review and editing; methodology; data curation; visualization. **Ting Niu**: Writing—original draft; investigation; methodology; data curation. **Judong Li**: Investigation; methodology; data curation. **Wei Niu**: Investigation. **Minghui Zheng**: Investigation. **Quanjiang Ji**: Resources. **Chuan Li**: Resources. **Chenghao Shao**: Project administration; supervision; resources; funding acquisition; writing—review and editing; conceptualization; validation. **Kan Ding**: Supervision; project administration; funding acquisition; conceptualization; validation; writing—review and editing. All authors have read the final manuscript and approved it for publication.

## Supporting information

Supporting File 1

Supporting File 2

## Data Availability

The data that support the findings of this study are available from the corresponding author upon reasonable request. The microbiota raw sequencing data of 16S rRNA gene sequencing of 135 clinical samples generated in this study have been uploaded to the NCBI Sequence Read Archive (SRA) database with the accession number PRJNA1190601 (https://www.ncbi.nlm.nih.gov/bioproject/PRJNA1190601/). The whole genome sequence of *E. hormaechei* A64 was deposited in the GeneBank with accession number CP103409 (https://www.ncbi.nlm.nih.gov/nuccore/CP103409.1). The accession number of 16S rRNA gene sequence of all the representative strains investigated in this study was also deposited in the GeneBank (Table S8). Supplementary materials (methods, figures, tables, graphical abstract, Chinese translated version, and updated materials) may be found in the online DOI or iMeta Science (http://www.imeta.science/).
