# Supplementary material for: Intratumoral Enterobacter hormaechei drives gemcitabine resistance in pancreatic cancer via cdd L‐mediated drug inactivation
Source: Imeta. 2026 Apr 29;5(2):e70126. doi: 10.1002/imt2.70126 (PMC13147963; doi:10.1002/imt2.70126)
Supplement: Supplementary file 1 — Supporting File 1 [file IMT2-5-e70126-s002.docx]

**Supporting information to**

**Intratumoral *Enterobacter hormaechei* drives gemcitabine resistance in pancreatic cancer via *cdd*_L_-mediated drug inactivation**

**Running title**: Bacteria in pancreatic cancer against chemosensitivity

Jun-Feng Peng^1,2,#^, Meixia Li^1,#,*^, Ting Niu^1,2,#^, Judong Li^1,2,#^, Wei Niu^3^, Minghui Zheng^1,2^, Quanjiang Ji^4^, Chuan Li^3,*^, Chenghao Shao^2,*^, Kan Ding^1,5,*^

*^1^Carbohydrate-Based Drug Research Center, CAS Key Laboratory of Receptor Research, State Key Laboratory of Drug Research, Shanghai Institute of Materia Medica, Chinese Academy of Sciences. Shanghai 201203, China.*

*^2^Department of Pancreatic-Biliary Surgery, Second Affiliated Hospital of Naval Medical University, Shanghai, 200003, China.*

*^3^State Key Laboratory of Drug Research, Shanghai Institute of Materia Medica, Chinese Academy of Sciences, Shanghai 201203, China.*

*^4^School of Physical Science and Technology & State Key Laboratory of Advanced Medical Materials and Devices, Shanghai Tech University, Shanghai 201210, China.*

*^5^Zhongshan Institute for Drug Discovery, Shanghai Institute of Materia Medica, Chinese Academy of Sciences, SSIP Healthcare and Medicine Demonstration Zone, Zhongshan 528400, China.*

^#^These authors contributed equally to this work: Jun-Feng Peng, Meixia Li, Ting Niu, Judong Li

^*^Correspondence: limei@simm.ac.cn (Meixia Li), chli@simm.ac.cn (Chuan Li), shaochenghao_czyy@163.com (Chenghao Shao), dingkan@simm.ac.cn (Kan Ding)

**METHODS**

**Clinical samples**

From 2017 to 2021, 71 pancreatic cancer patients were collected by the Second Affiliated Hospital of Naval Medical University (Shanghai, China), and fresh pancreatic cancer and paracancerous tissues were sampled, and stored at the Second Affiliated Hospital of Naval Medical University sample library at –80 °C. Detail information on the 71 pancreatic cancer patients, including patient ID (identity document, ID), gender, age, TNM, stage, and collection time is listed in Table S1. A total of 142 samples, including pancreatic cancer and paracancerous tissues, were used for diversity sequencing based on the entir 16S rRNA gene of the bacteria. Considering that these samples were fresh for bacterial isolation, fresh clinical samples were collected again by the Second Affiliated Hospital of Naval Medical University (Shanghai, China) from 2021 to 2022. Briefly, fresh pancreatic cancer and paracancerous tissues were sampled immediately with fresh sterile DMEM for bacteria isolation. A total of 43 patients with pancreatic cancer including 86 samples, were used for bacterial isolation and identification. Detailed information on 43 patients with pancreatic cancer, including patient ID (identity document, ID), gender, age, TNM, stage, and collection time are shown in Table S4.

**16S rRNA gene sequencing and data analysis**

Although 142 samples were collected for 16S rRNA gene sequencing, 7 samples did not satisfy the sequencing conditions. Thus, a total of 135 samples including pancreatic cancer tissues 75 and adjancet tissues 60 were preapared for 16S rRNA gene sequencing. The DNA library was prepared using the primer pair 27F (5’-AGRGTTYGATYMTGGCTCAG-3’) and 1492R (5’-RGYTACCTTGTTACGACTT-3’) targeting the full-length of the 16S rRNA gene and sequenced on a Pacbio Sequel IIe System (Pacific Biosciences) by Majorbio Bio-Pharm Technology Co. Ltd. (Shanghai, China). Data were processed using SMRTLink (v.11.0) to obtain high-fidelity (Hifi) reads that were de-noised using the DADA2 plugin in Qiime2 to obtain amplicon sequence variants (ASVs). Taxonomic assignment was accomplished using a vsearch taxonomic classifier and annotated using the Silva 16S rRNA database (v.138). The analyses were performed using the Majorbio Cloud Platform (https://cloud.majorbio.com), and the details of the database and software are listed in Table S14. Notably, to minimize environmental contamination, strict aseptic techniques were maintained throughout the study. For 16S rRNA gene sequencing, DNA extraction negative controls (sterile water and buffer) and PCR negative controls were included in every batch. Sequence variants (ASVs) present in negative controls were identified as contaminants and bioinformatically removed from the dataset using the decontam R package.

**Intratumor bacteria isolation and identification**

To isolate bacteria from the intratumor, four kinds of agar culture-medium including Fastidious Anaerobe Broth (FAB, Cat: YYMD045, Shanghai Haling Biological Technology Co., LTD, China), Bold’s Basal Medium (BBM, Sigma, USA), Yeast extract, Casitone, and Fatty Acid (YCFA, Cat: YYMD046, Shanghai Haling Biological Technology Co., LTD, China) and Gifu Anaerobic Medium (GAM, Cat: YYP1875, Shanghai Haling Biological Technology Co., LTD, China) supplemented with 5% sterile defibrillated sheep blood, respectively, were emplored to do bacteria isolation. In brief, fresh tumor tissues underwent a surface sterilization procedure involving immersion in 75% ethanol for 30 seconds followed by three washes in sterile PBS to eliminate surface adherents. Only the inner core of the tissue was used for homogenization and plating, ensuring that the isolates represented bona fide intratumoral residents. Next, the pancreatic cancer and adjacent tissues were homogenized and spread in four kinds of agar culture medium, respectively, and cultured in an anerobic incubator at 37 °C for 24–72 h. Then, a single colony was picked and cultured in the corresponding agar plates for another 24–72 h at 37 °C in an anaerobic incubator. The colonies grown on the plate were washed with liquid medium and separated into two parts. One part was used to prepare the glycerol stocks, and the other was used to perform DNA extraction and 16S rRNA gene amplification. The PCR products were tested using agarose gel electrophoresis and subsequently sequenced (Sangon, China). For some representative strains, the entire 16S rRNA gene sequence was amplified, and the sequence accession number was deposited in the National Center for Biotechnology Information (NCBI) database. The primers used for bacterial identification are listed in Table S15.

**Histology analysis**

Pancreatic cancer tissues were fixed in 4% paraformaldehyde, embedded in paraffin, and cut into 5 μm sections. Bacteria were detected by immunohistochemistry (IHC) using an anti-mouse polyclonal antibody. The antibodies used in this study are listed in Table S16.

**High-performance liquid chromatography-tandem mass spectrometry**

*E. hormaechei* A64, *C. acnes* (B16)*, S. epidermidis* (B13)*, K. pneumoniae* (B80), *P. aeruginosa* (C9), *Bacillus cereus* (C4)*, Lacrimispora sphenoides* (C25) and *Citrobacter freundii* (B79) were recovered with FAB medium in anerobic incubator at 37 °C for 12 h. All species were passaged in FAB medium in an anaerobic incubator at 37 °C. The cells were then collected and washed once with 0.9% NaCl, and the OD_600_ value of all the species was adjusted around 0.4 by FAB medium. Meanwhile, 10 mM gemcitabine was diluted to 1 mM using the sterile Millipore water. Finally, 50 µL of diluted gemcitabine and 50 µL diluted species were added to a 1.5 mL sterile centrifuge tube and cultured at 37 °C in an anaerobic incubator. Every 1 h, three duplicate samples were taken out, and the supernatants were collected by centrifugation and stored at –20 °C. Before detection, 50 µL of the supernatant was added to triple the volume methanol, followed by shock (1,600 rpm, 5 min) and centrifugation (16,000 rpm, 5 min) to precipitate the proteins. Finally, 50 µL supernatants were collected to do LC-MS/MS analysis.

To determine the levels of gemcitabine (dFdC) and inactive gemcitabine (dFdU) in the supernatants, an LC–MS/MS instrument consisting of an Agilent 1290 Infinity II (SIMM-HM/DMPK-132, Germany) and an API4000 Q trap (SIMM-HM/DMPK-003, Germany) was used. 1290 II and Analyst are the two software packages used in this system. The conditions of chromatography and mass spectrometry are listed in Table S17.

**Analysis of enzymatic activity of CDD_L_**

The enzymatic activity of CDD_L_ is detected using Liquid Chromatography-Mass Spectrometry/Mass Spectrometry method for metabolite concentration analysis described as previously report [1]. First establish incubation conditions that allow metabolites to form linearly over incubation time. Determine the optimal incubation time and protein concentration to ensure the probe substrate loss rate remains below 20% throughout the reaction. The final concentration of CDD_L_ selected was 0.7 μM, which was incubated with gemcitabine at concentrations ranging from 50 to 800 μM for 1 min at 37°C in the buffer (pH = 7.5) consisted of 50 mM Tris-HCl, 100 mM KCl, and 10% Glycerol. Subsequently, the concentration of the generated *2*',*2*'-difluorodeoxyuridine (dFdU) was measured.

**Cell culture**

Pancreatic cancer cell lines, including PANC-1 (Ref. SCSP-535), CFPAC-1(Ref. TCHu112), BxPC-3 (Ref. TCHu 12) and MiaPaCa-2 (Ref. SCSP-568) were purchased from the Cell Bank of the Type Culture Collection Center of the Chinese Academy of Sciences (Shanghai, China). The PANC-1 and MiaPaCa-2 cell lines were cultured in Dulbecco's Modified Eagle's medium (DMEM) (Cat: 12430054, Gibco, USA) supplemented with 10% fetal bovine serum (FBS) (Cat: A5669701, Gibco, USA), 100 U/mL penicillin and 100 mg/mL streptomycin. CFPAC-1 and BxPC-3 cells were cultured in RPMI-1640 medium supplemented with 10% fetal bovine serum (FBS, Cat: A5669701, Gibco, USA), 100 U/mL penicillin, and 100 µg/mL streptomycin. Incubation was conducted in a humidified environment containing 5% CO₂ at 37 °C.

**Animal experiments**

Eight-week-old BALB/c nu/nu mice weighing 20 ± 2 g were purchased from Beijing Viton Lever Laboratory Animal Technology Co, Ltd and housed in the specific pathogen-free (SPF) facilities of the Experimental Animal Centre of the Shanghai Institute of Material Medica, Chinese Academy of Sciences. Subcutaneous graft tumor formation by subcutaneous injection of PDAC cells or autologous tumor block transplantation in mice. Tumor growth was monitored twice a week after inoculation, and body weight and tumor size of the nude mice were recorded. When palpable tumors grew under the skin (generally around 100-150 mm^3^), body weight of nude mice and the long and short diameters of their subcutaneous tumors were measured, and the tumor volume was calculated. Nude mice were grouped according to the tumor volume and body weight using a stratified random sampling method to ensure that body weight and tumor volume were balanced and not significantly different between the groups before administration.

**Animal Experiment 1:** The function of *E. hormaechei* A64 on gemcitabine

Nude mice were subcutaneously inoculated with MIA PaCa-2 cells (5 × 10^6^ cells/100 μL) to establish a transplant tumor model. After tumor formation, the mice were grouped according to body weight and transplanted tumor volume into four groups, including: (1) Control group, mice in control group were injected intraperitoneally with the same volume of normal saline as in the gemcitabine group; (2) Gemcitabine (Gem) treatment group, mice in this group were intraperitoneal injections of gemcitabine (50 mg/kg) given on days 20 and 23, and intraperitoneal injections of gemcitabine (150 mg/kg) were given at 4 days intervals starting on day 26 after inoculation; (3) *E. hormaechei* A64 inoculation group, mice in this group were injected into the tumor on days 19 and 29 after tumor inoculation with 4×10^8^ CFU/mL *E. hormaechei* A64; (4) *E. hormaechei* A64 + Gem treatment group, mice in this group were injected into the tumor on days 19 and 29 after tumor inoculation with 4×10^8^ CFU/mL *E. hormaechei* A64. Intraperitoneal injections of gemcitabine (50 mg/kg) were given on days 20 and 23, and intraperitoneal injections of gemcitabine (150 mg/kg) were given at 4 days intervals starting on day 26 after inoculation. In the endpoint, all mice were sacrificed, and the tumors were collected.

**Animal Experiment 2:** The function of *E. hormaechei* A64_Δ*cdd*_L_ on gemcitabine

Nude mice were subcutaneously inoculated with MIA PaCa-2 cells (5 × 10^6^ cells/100 μL) to establish a transplant tumor model. After tumor formation, the mice were grouped according to body weight and transplanted tumor volume into four groups, including: (1) Contro group, mice in the control group were injected intraperitoneally with the same volume of normal saline as in the gemcitabine group; (2) Gem treatment group, mice in this group was intraperitoneal injections of gemcitabine (50 mg/kg) were given on days 20 and 23, and intraperitoneal injections of gemcitabine (150 mg/kg) were given at 4 days intervals starting on day 26 after inoculation; (3) *E. hormaechei* A64_Δ*cdd*_L_ inoculation group, mice in this group were injected into the tumor on days 19 and 29 with 4 × 10^8^ CFU/mL *E. hormaechei* A64_Δ*cdd*_L_, after tumor inoculation; (4) Gem + *E. hormaechei* A64_Δ*cdd*_L_ group, mice in this group were injected into the tumor on days 19 and 29 with 4 × 10^8^ CFU/mL *E. hormaechei* A64_Δ*cdd*_L_, after tumor inoculation. Meanwhile, mice were intraperitoneal injections of gemcitabine (50 mg/kg) were given on days 20 and 23, and intraperitoneal injections of gemcitabine (150 mg/kg) were given at 4-day intervals starting on day 26 after inoculation. In the endpoint, all mice were sacrificed, and the tumors were collected.

**Animal Experiment 3:** The colonization of *E. hormaechei* A64 in the tumor

The vector pGEN-luxCDABE was transformed into the *E. hormaechei* A64 by electro transformation yield a A64_pGEN-luxCDABE strain. Then, the overnight culture of A64_pGEN-luxCDABE was mixed with BxPC-3 cells, and the mixture was injected into both the pancreas in situ (2×10^6^ cells/50 μL) and the right dorsal subcutaneous (4×10^6^ cells/100 μL) of the nude mice, respectively. In contrast, the mixture of *E. hormaechei* A64 and BxPC-3 cells as control were also injected into the same place of nude mice. The fluorescence was visualized by a Small Animal In Vivo Imaging System (IVIS^®^ Spectrum). In the endpoint, all mice were sacrificed, and the tumors were collected.

**Animal Experiment 4:** Gemcitabine in combination with antibiotic

Nude mice were subcutaneously inoculated with MIA PaCa-2 cells (5×10^6^ cells/100 μL) to establish a transplant tumor model. After tumor formation, mice were grouped according to the body weight and transplanted tumor volume into six groups, including: (1) Control group, mice in this group were injected intraperitoneally with the same volume of normal saline as in the gemcitabine group. (2) 150 mg/kg or 75 mg/kg Gem treatment group, mice in these two groups were intraperitoneal injections of 150 mg/kg or 75 mg/kg gemcitabine; (3) 150 mg/kg Gem + A64 group, mice in this group were injected into the tumor on days 12 after tumor inoculation with 4 × 10^8^ CFU/mL *E. hormaechei* A64, and intraperitoneal injections of gemcitabine (150 mg/kg) were given at 6-day intervals starting on day 15 after inoculation. (4) 150 or 75 mg/kg Gem + *E. hormaechei* A64 + Cefe group, mice in these groups were injected into the tumor on days 12 after tumor inoculation with 4 × 10^8^ CFU/mL *E. hormaechei* A64, and intraperitoneal injections of gemcitabine (150 or 75 mg/kg) were given at 6-day intervals starting on day 15 after inoculation. Meanwhile, mice in these two groups were oral gavage with 300 mg/kg cefepime every day from day 15 to 21. In the endpoint, mice were sacrificed, and the tumors were collected for bacterial isolation.

**Bacterial conditions medium preparation**

The day before, the pancreatic cancer cell lines including, PANC-1, CFPAC-1, BxPC-3, and MiaPaCa-2 were digested and seeded in 96-well plates (~2,000 cells per well). Overnight cultures of *E. hormaechei* A64) in FAB medium were collected, washed once with 0.9% NaCl, and then resuspended in DMEM medium to adjust the OD_600_ value to 0.4. Gemcitabine (10 mM) was diluted to 1 mM in DMEM. Diluted *E. hormaechei* A64 and gemcitabine were then mixed with an equal volume and cultured at 37 °C in an aerobic incubator for 6–8 h. The supernatants were collected by centrifugation, and filtered using a 0.22 µm filter and diluted 500 fold with DMEM. Then, 100 µL of the filtered medium was added to the 96-well plates and incubated with the pancreatic cancer cell lines for another 72 h. Finally, cell viability was measured using the CCK-8 method, according to the manufacturer’s instructions.

**Polyclonal antibody preparation**

Polyclonal antibodies were prepared as described previously [2]. In brief, 600 μg/mL purified recombinant proteins or 10^10^ CFU/mL bacteria were mixed with an equal volume of Freund’s complete adjuvant (Sigma-Aldrich, St. Louis, MO, USA). Subsequently, a mixture of proteins or bacteria was injected subcutaneously multipoint into 6-week-old BALAB/c mice. Two weeks after the first injection, the mice were immunized twice at 1-week interval, using the same amounts of proteins or bacteria which were mixed with an equal volume of Freund’s incomplete adjuvant (Sigma-Aldrich, St. Louis, MO, USA). Blood was obtained 7 days after the last injection and was stored at room temperature for 1 h. The supernatants were collected by centrifugation at 2200 rpm for 20 min and then stored at –80 °C.

***In vitro* labeling of bacteria with FADA**

Bacteria were labeled with 0.2 mM FADA probes in the dark for 5 h of incubation in FAB medium. Labeled bacteria were collected by centrifugation. Fluorescence was visualized using a laser scanning confocal microscope FV1000 (Olympus FV1000-SIM, Japan).

**Western blotting**

*E. hormaechei* A64 and *E. hormaechei* A64_Δ*cdd*_L_ strains were cultured, respectively, in anaerobic incubator at 37 °C for overnight. Following, the cells were collected by centrifugation and washed once using 0.9% NaCl. Total bacterial proteins were obtained by repeated freeze-thawing twice, and then 5 × loading buffers were added and heated at 100 °C for 10 min. Next, 10 µL of protein was loaded and separated by 12% SDS-PAGE gel. The separated proteins were then transferred to the nitrocellulose (NC) membranes (Millipore, USA). Membranes were sequentially blocked with 1 × TBST containing 5% BSA (*w*/*v*) and incubated with primary antibodies including mouse-anti-CDD_L_ (1: 1000, *v*/*v*) or mouse-anti-RpoB (1: 1000, *v*/*v*) overnight at 4 ℃. After 3 times washing with 1 × TBST, the membranes were sequentially incubated with the second antibody (goat-anti-mouse, 1: 5000) for 1 h at room temperature, followed by washing with 1 × TBST three times. Finally, the bands were visualized using enhanced chemiluminescence (ECL) reagents (Thermo, USA) and ImageQuantLAS 4000 (GE, USA).

**Subcloning and protein purification**

The open-reading frames encoding mature forms of the *cdd*_L_ and *rpoB* used in this study were ampliﬁed from the genomic DNA of *E. hormaechei* A64 by PCR which introduced BamHI/XhoI sites into the ﬂanks of the target genes. The ampliﬁed DNA was cloned into the expression vector pET28a and transformed into Tuner (DE3) competent cells for protein expression. The recombinant protein contained an N-terminal His 6-tag. The tuner (DE3)-containing vector was cultured in Luria-Bertani (LB) broth containing kanamycin (50 µg/mL) at 37 °C. Cells were grown to mid-log phase (OD_600_ ~ 0.6), at which point isopropyl β-D-thiogalactopyranoside (IPTG) was added to a ﬁnal concentration of 0.5 mM, and the cultures were shaken (200 rpm) for a further 16 h at 16 °C. The cells were harvested by centrifugation and sonicated, and His 6-tagged recombinant protein was puriﬁed. Site-directed mutants of E104A were generated using a Quikchange Kit (Agilent). The buffers for protein purification used in this study are listed in Table S18. Primers used for cloning and mutation are listed in Table S15.

**Bacterial 16S rRNA fluorescence in situ hybridization (FISH)**

Ribosomal RNA FISH was performed for the visualization of bacteria in human solid tumor types including breast, lung, melanoma, esophageal and ovarian cancers. The protocol was adapted from previous publication and optimized for formalin-fixed paraffin-embedded (FFPE) tissues [3]. We designed a probe complementary to the 16S rRNA region of *Enterobacter* spp. The sequence of probe is ATCGCTCTGCTCCGCCGCCA.

**Statistical analysis**

GraphPad Prism 10.1 software (San Diego, CA, USA) was used to perform all statistical analyses and create all pictures. Image combination was performed using Adobe Illustrator 2020 software. Figure 1A & Figure 1C were prepared using BioRender online (https://www.biorender.com/). All data are presented as the mean ± SD. For statistical comparisons, normally distributed variables across multiple groups were analyzed using one-way analysis of variance (ANOVA), followed by Tukey’s *post hoc* test (assuming homogeneity of variances) or Dunnett’s T3 test (assuming unequal variances). Non-normally distributed data were compared using the Mann-Whitney U test for two independent groups, and the Kruskal–Wallis test for multiple groups. For 16S rRNA sequencing data, *p*-values were adjusted for multiple testing using the false discovery rate (FDR) method. Longitudinal repeated-measures data, including tumor volume growth curves over time, were analyzed using two-way repeated-measures ANOVA to account for group-by-time interactions and potential missing values. This was followed by Tukey's *post hoc* multiple comparisons test to evaluate statistical differences between specific treatment groups at the indicated time points. Statistical significance was set at *p* < 0.05.


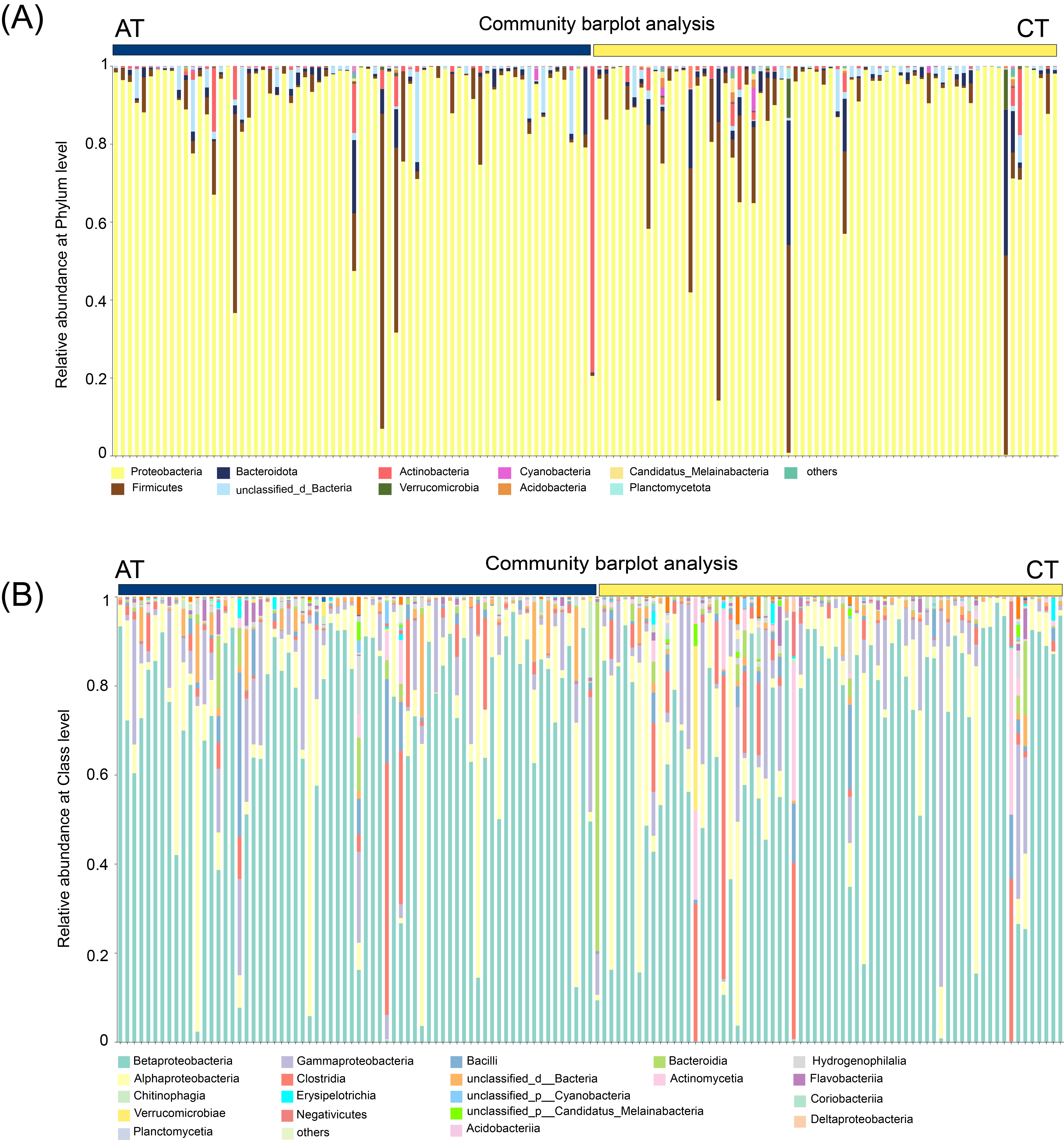


**Figure S1 Taxonomic composition of the pancreatic tumor microbiome**. (A) Stacked bar plots showing the relative abundance of bacterial flora at the phylum level in paired adjacent tissues (AT, *n* = 67) and cancer tissues (CT). *Proteobacteria* (yellow) were the predominant phylum across the cohort. (B) Relative abundance of bacterial taxa at the class level. The community structure was dominated by *Betaproteobacteria* (teal) and *Gammaproteobacteria* (purple). Each column represents an individual sample (*n* = 135 samples from 71 patients).





**Figure S2** **Intratumor bacteria were explored by 16S rRNA gene sequencing.** α diversity sequencing (A) Ace index (B) Chao index, and (C) Shannon index. (D) β diversity sequencing at the ASV level. *P* value was calculated by ANOSIM. (E) Venn analysis between AT (*n* = 60) and CT (*n* = 75) at genus level. *P*-value of α diversity sequencing was calculated by the Mann-Whitney U test for two independent groups.


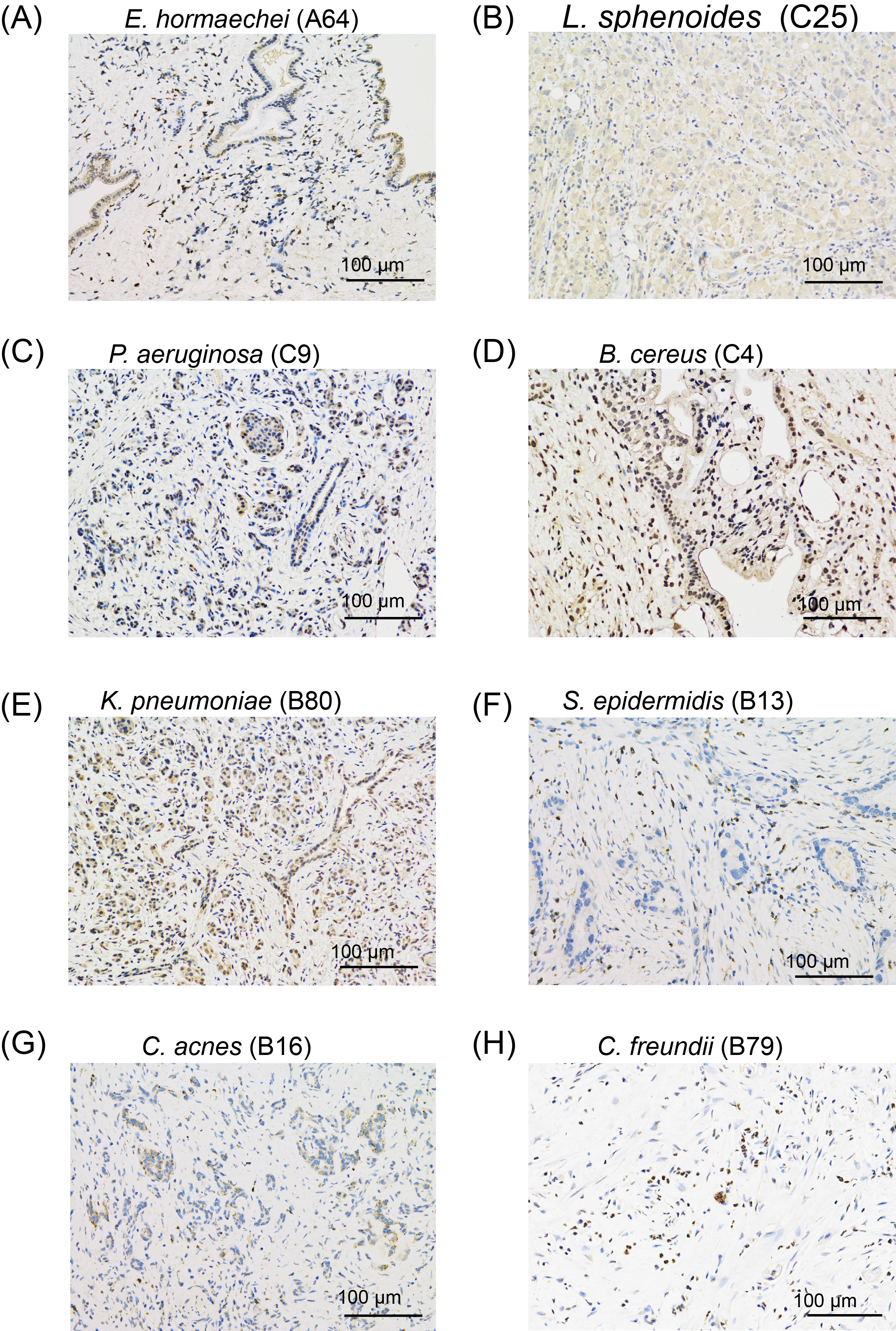


**Figure S3 Pancreatic cancer tissue slides are stained by immunohistochemical (IHC) using an anti-bacterial polyclonal antibody.** (A) Anti-*E. hormaechei* (A64). (B) Anti-*L. sphenoides* (C25). (C) Anti-*P. aeruginosa* (C9). (D) Anti-*B. cereus* (C4). (E) Anti-*K. pneumoniae* (B80). (F) Anti-*S. epidermidis* (B13). (G) Anti-*C. acnes* (B16). (H) Anti-*C. freundii* (B79).


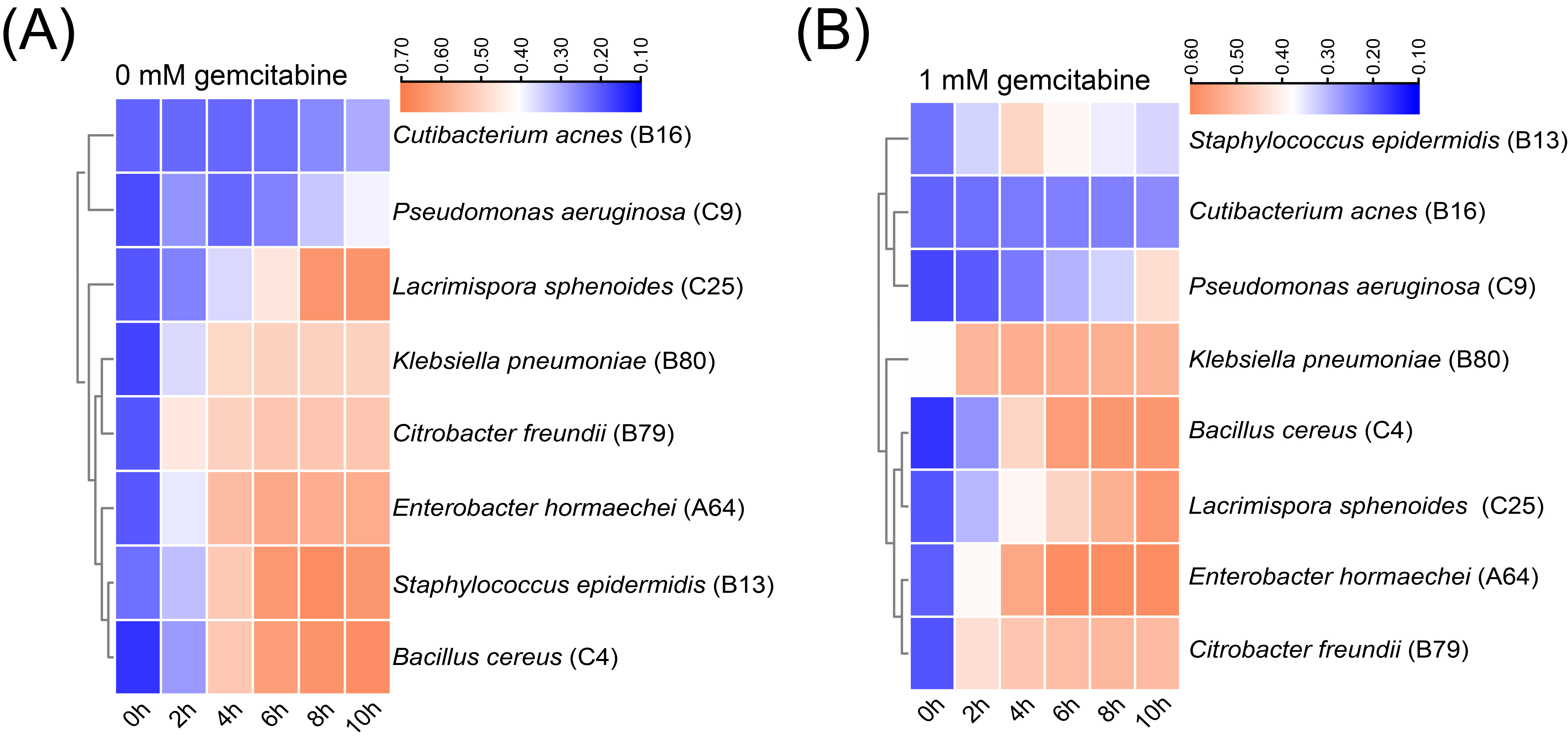


**Figure S4 The growth of clinically isolated bacteria in FAB medium with or without gemcitabine is shown by a heatmap.** (A) Clinical isolates grown without gemcitabine. (B) Clinical isolates grown under 1 mM gemcitabine.


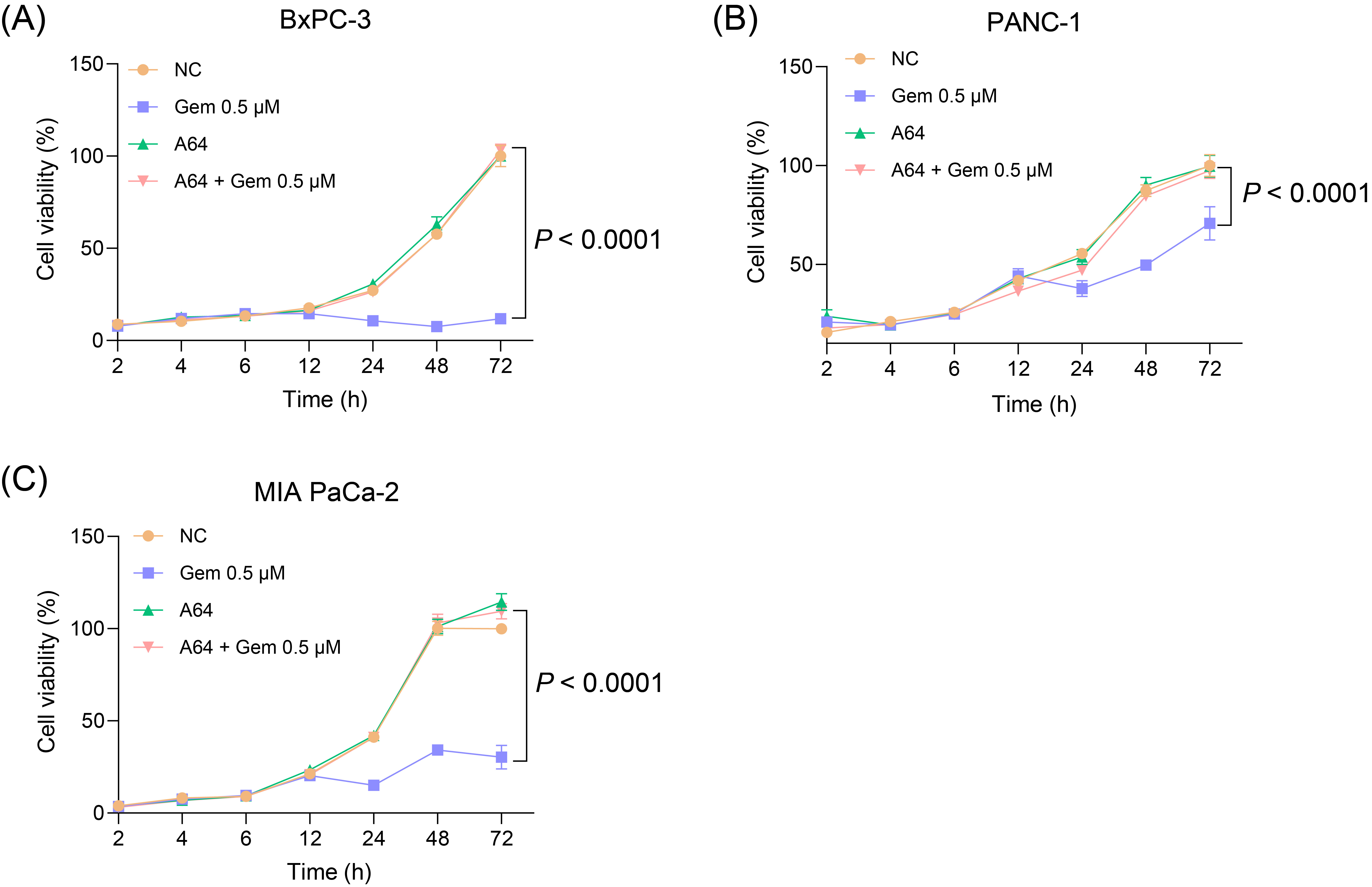


**Figure S5**. ***Enterobacter hormaechei* (A64) confers gemcitabine resistance in pancreatic cancer cell lines.** Time-dependent cell viability assays were performed in (A) BxPC-3, (B) PANC-1, and (C) MIA PaCa-2 cells. Cells were treated with gemcitabine (Gem, 0.5 μM) in the presence or absence of *E. hormaechei* (A64) co-culture. The negative control (NC) group received PBS. Bacterial colonization (A64 + Gem group, pink line) significantly abrogated the cytotoxic effect of gemcitabine compared to the gemcitabine-only group (purple line). Data are presented as mean ± SD. Statistical significance was determined using two-way ANOVA.

**
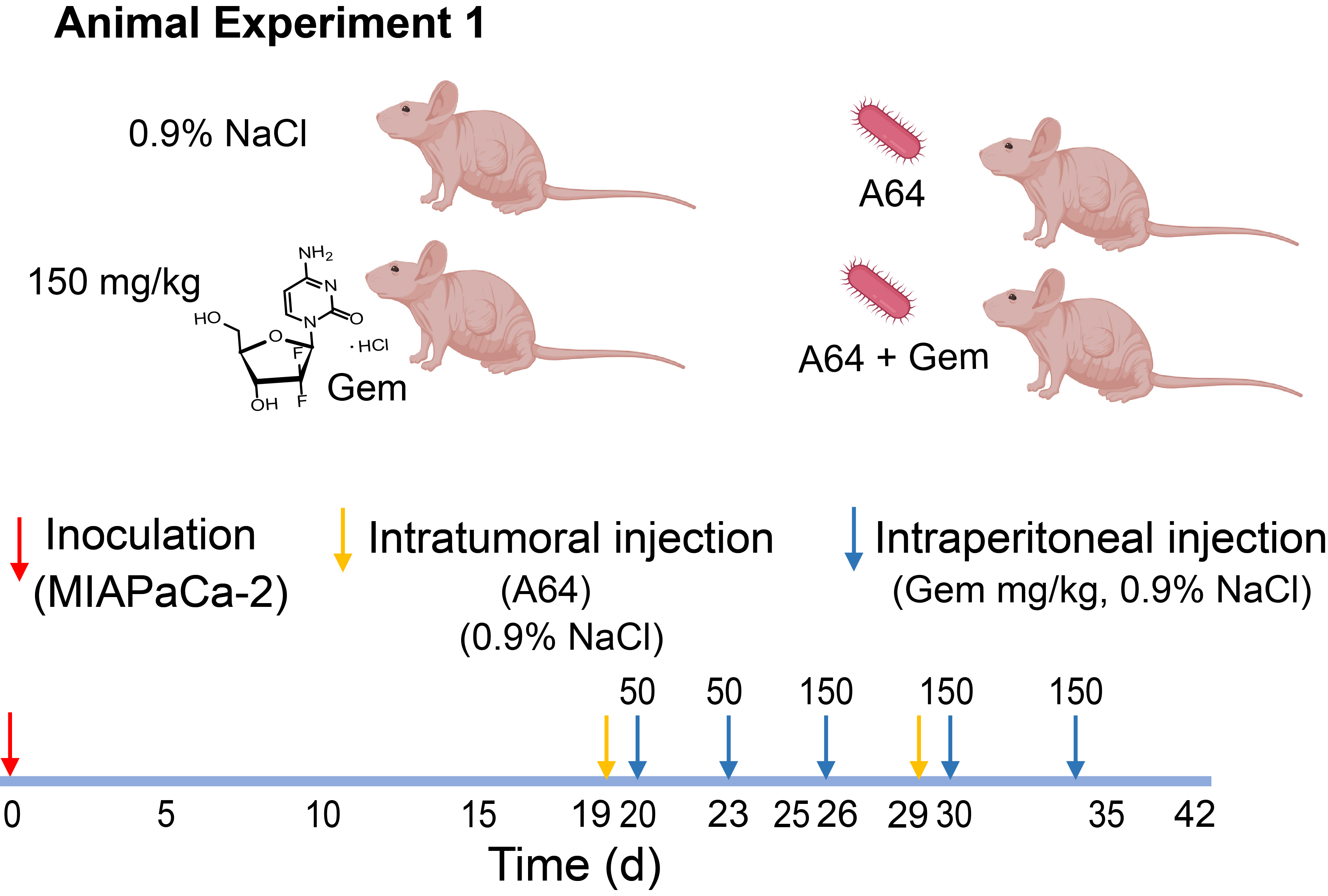
**

**Figure S6 The schematic of animal experiment 1**. Nude mice were subcutaneously inoculated with MIA PaCa-2 cells (5 × 10^6^ cells/100 μL) to establish a transplant tumor model.


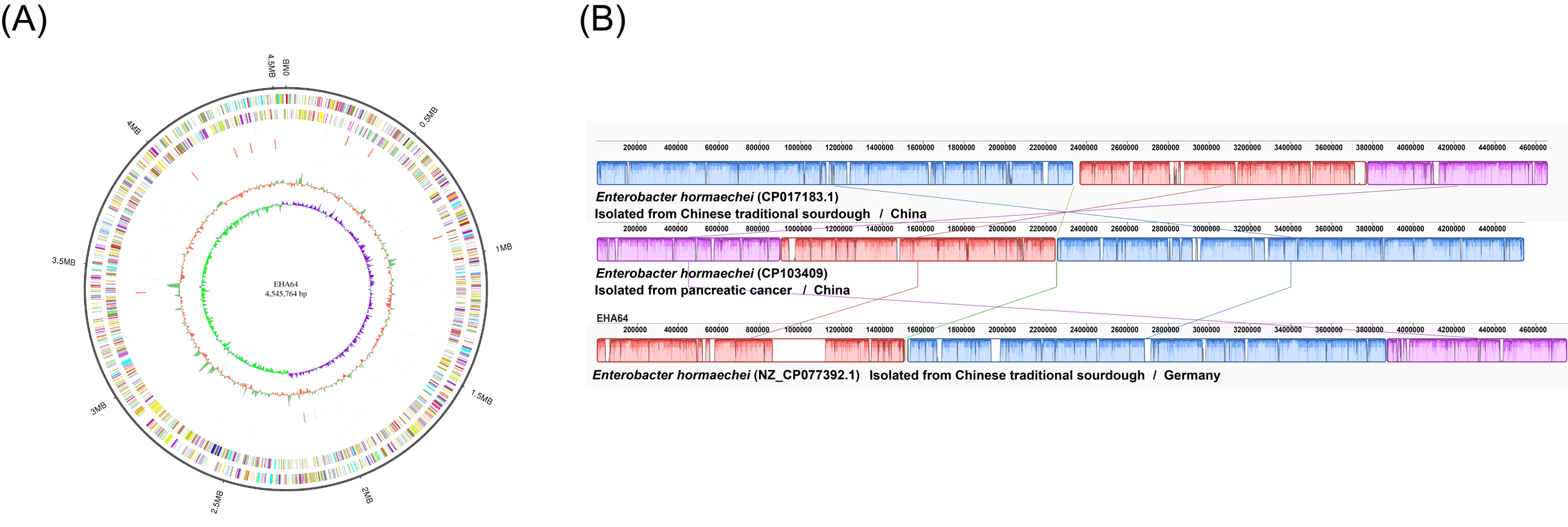


**Figure S7 Genomic sequencing of *Enterobacter hormaechei* A64**. (A) Genome map of *E. hormaechei* (A64). (B) Genome comparation among *E. hormaechei* strains.


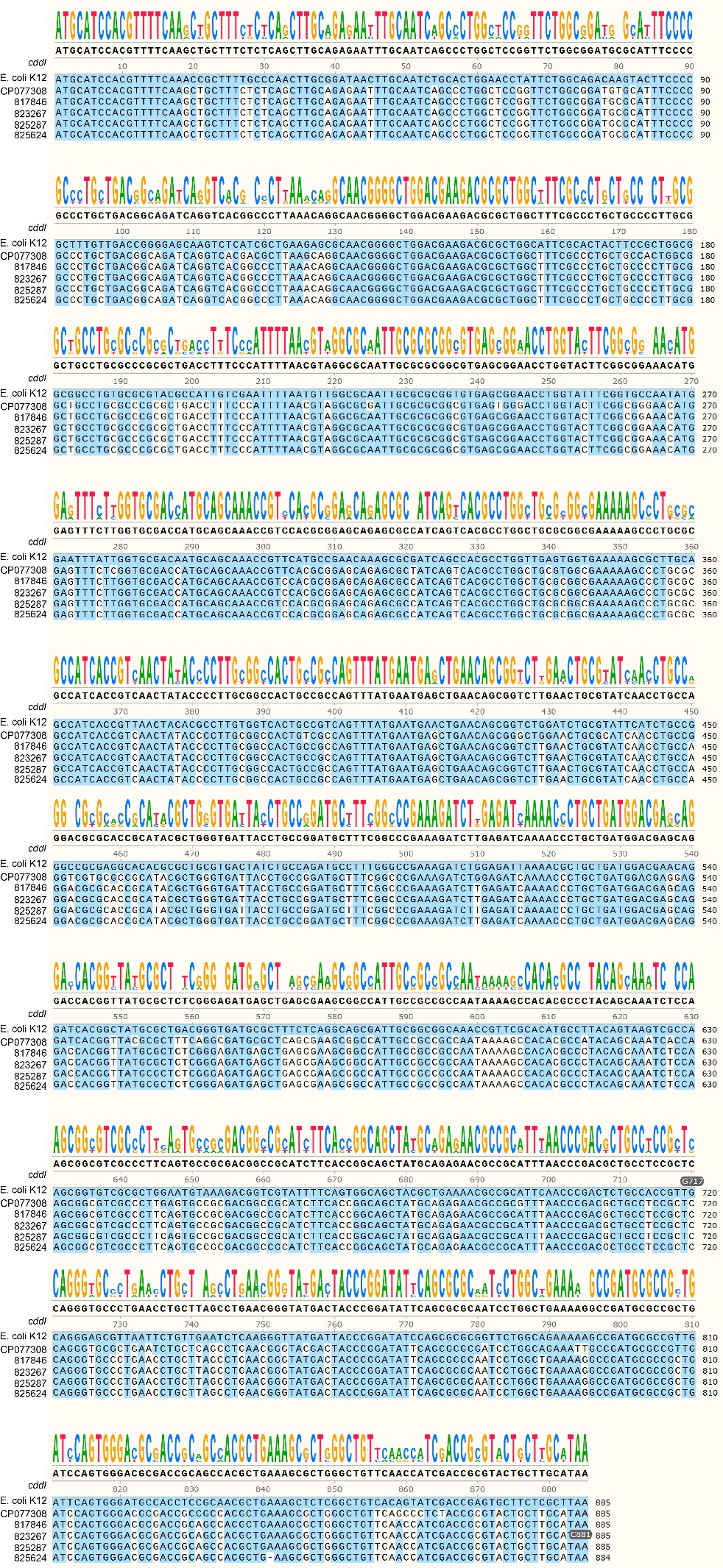


**Figure S8 Nucleotide sequence alignment of the *cdd*_L_ gene.** Alignment of the nucleotide sequence of *cdd*_L_ from the genome of *E. coli* and the clinical isolates *E. hormaechei*, respectively.


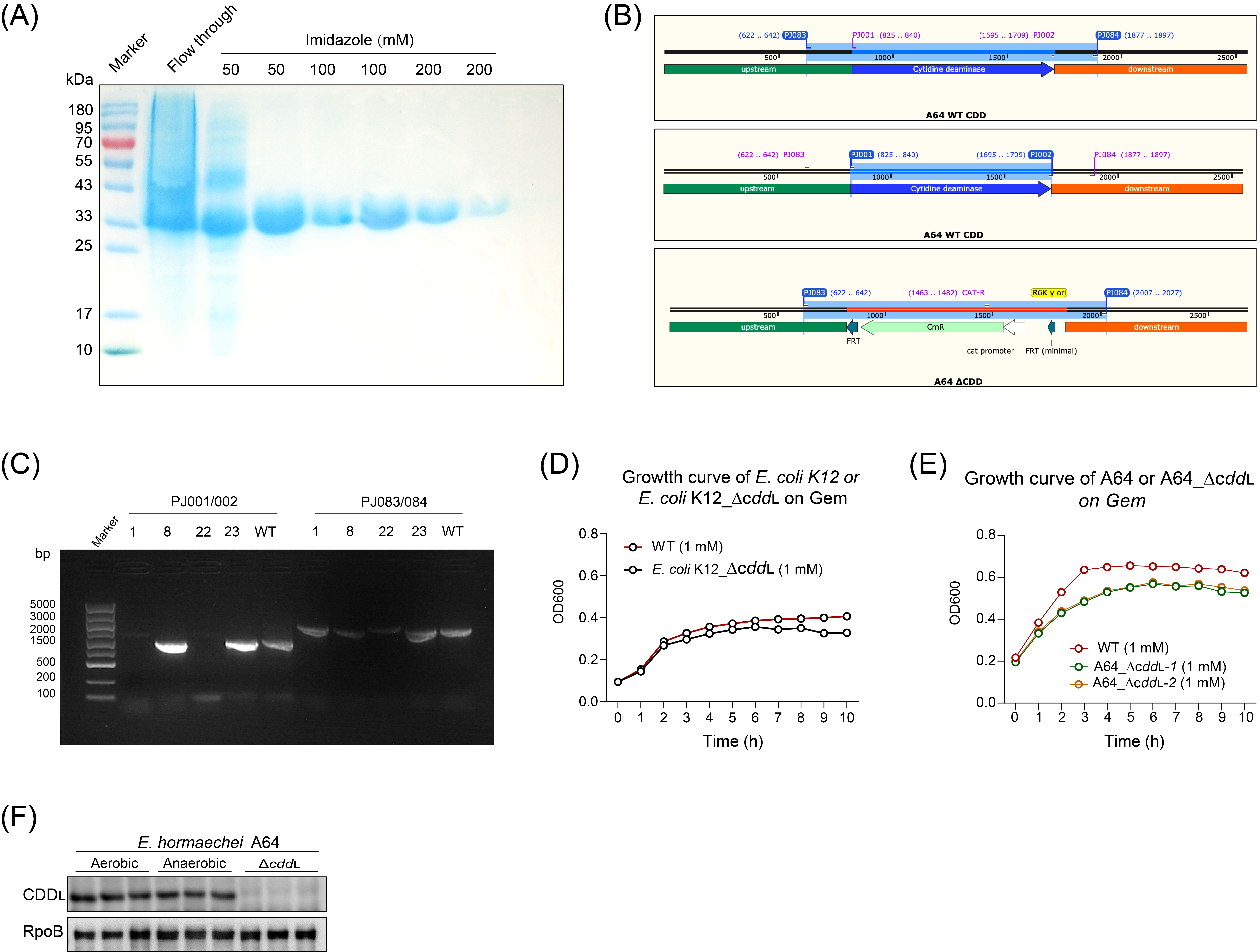


**Figure S9 Expression and purification of the CDD_L_ protein, and construction of the A64_*cdd*_L_-deficient strain.** (A) CDD_L_ protein expression using BL21(DE3) cells. (B) Schematic diagram of A64_Δ*cdd*_L_ deficient strain construction. (C) PCR identification. (D) Growth curve of *E. coli* K12 or *E. coli* K12_Δ*cdd*_L_ strain on 1 mM gemcitabine. (E) Growth curve of A64 or A64_Δ*cdd*_L_ strain on 1 mM gemcitabine. (F) The expression of CDD_L_ was detected by Western blotting.


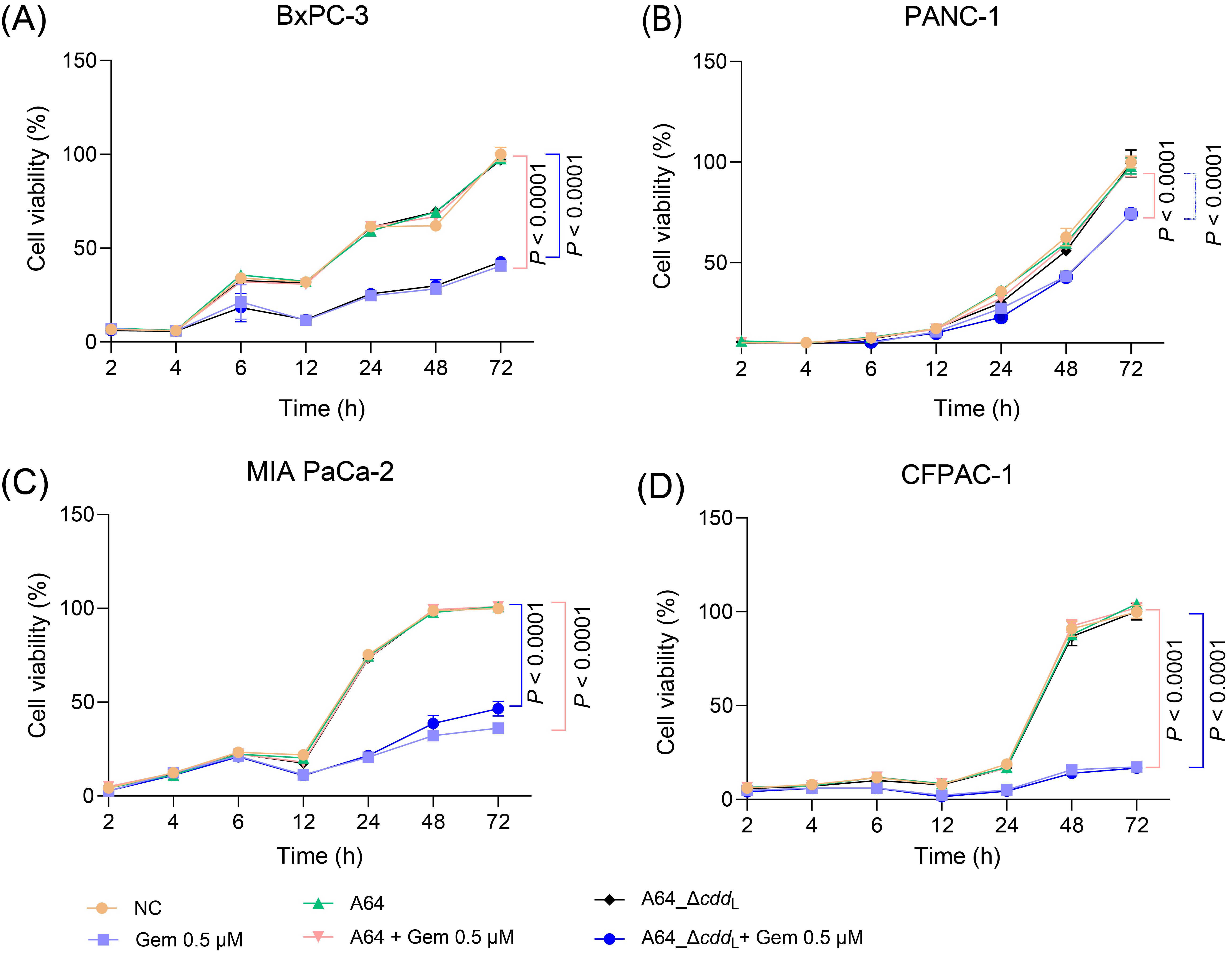


**Figure S10** **Genetic ablation of the *cdd*_L_ gene abolishes *E. hormaechei*-mediated gemcitabine resistance *in vitro*.** Time-dependent cell viability assays were performed in (A) BxPC-3, (B) PANC-1, (C) MIA PaCa-2, and (D) CFPAC-1 pancreatic cancer cell lines. Cells were treated with gemcitabine (Gem, 0.5 μM) in the presence of PBS (NC), wild-type *E. hormaechei* (A64), or the *cdd*_L_-knockout mutant (A64_Δ*cdd*_L_). While co-culture with WT A64 (pink line) significantly protected tumor cells from gemcitabine-induced cytotoxicity, this protective effect was completely abrogated in the group co-cultured with the A64_*cdd*_L_ mutant (dark blue line). The cell viability of the mutant group remained comparable to that of the Gem-only treatment (purple square), confirming that gemcitabine resistance is strictly dependent on the *cdd*_L_ gene. Data are presented as mean ± SD of three independent experiments. Statistical significance was determined using two-way ANOVA. *p* < 0.0001.


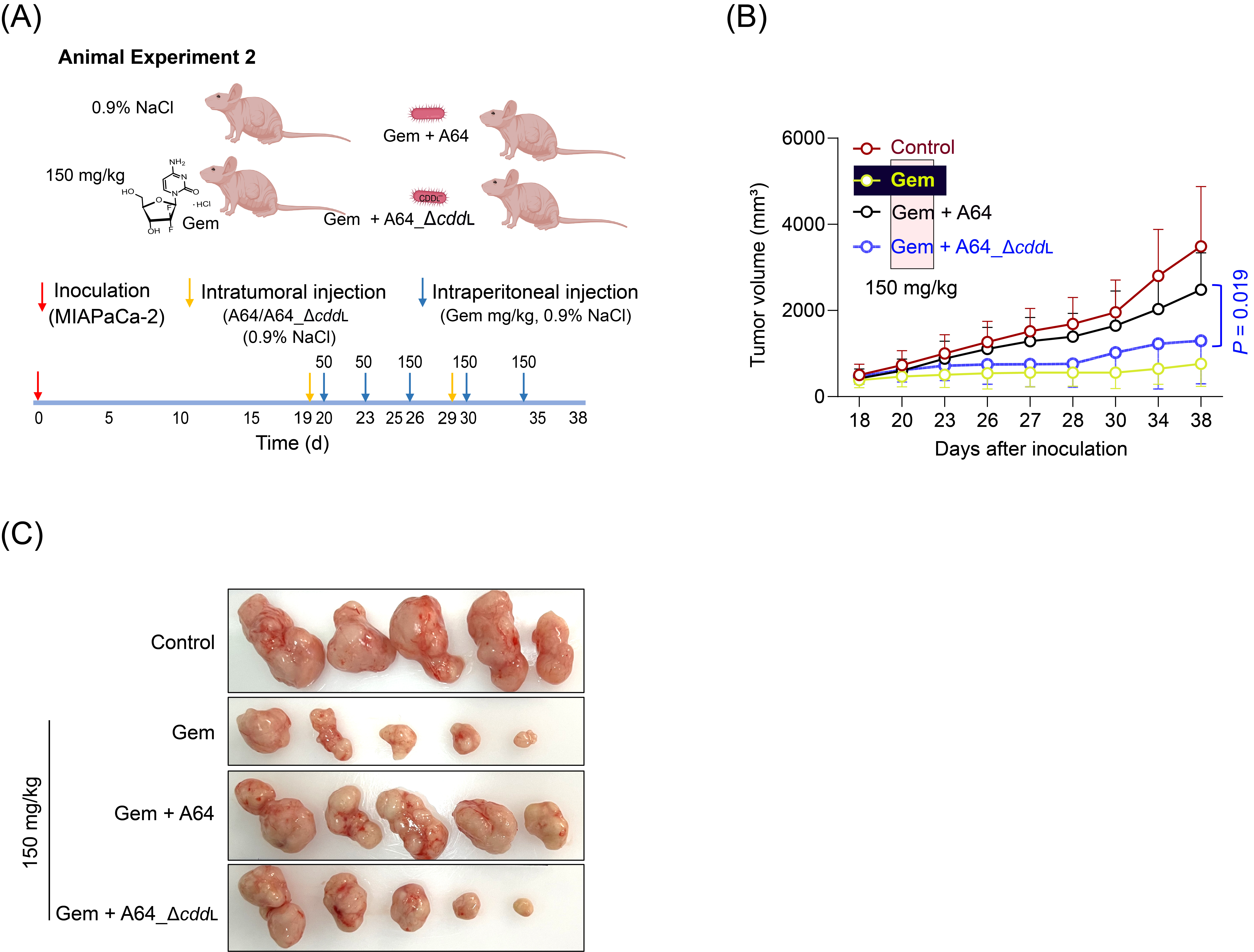


**Figure S11 *E. hormaechei* A64_Δ*cdd*_L_ reverses gemcitabine resistance *in vivo*.** (A) Schematic diagram of Animal Experiment 2 (*n* = 5). Nude mice were inoculated with 5 × 10^6^ MIA-PaCa-2 cells in subcutaneous tissue on day 0. On days 19 and 29 after inoculation, mice were intratumorally injected with 4 × 10^7^ CFU/mL *E. hormaechei* A64 or *E. hormaechei* A64_Δ*cdd*_L_, respectively. On days 20 and 23 after inoculation, mice were intraperitoneally injected with 50 mg/kg gemcitabine. On days 26, 30, and 35 after inoculation, the mice were intraperitoneally injected with 150 mg/kg gemcitabine. (B) Tumor volume in each group was recorded at different time points (*n* = 5) (*P* = 0.019). (C) Morphology and size of the tumor were observed on day 38 (*n* = 5). *P* values were calculated by two-way ANOVA.


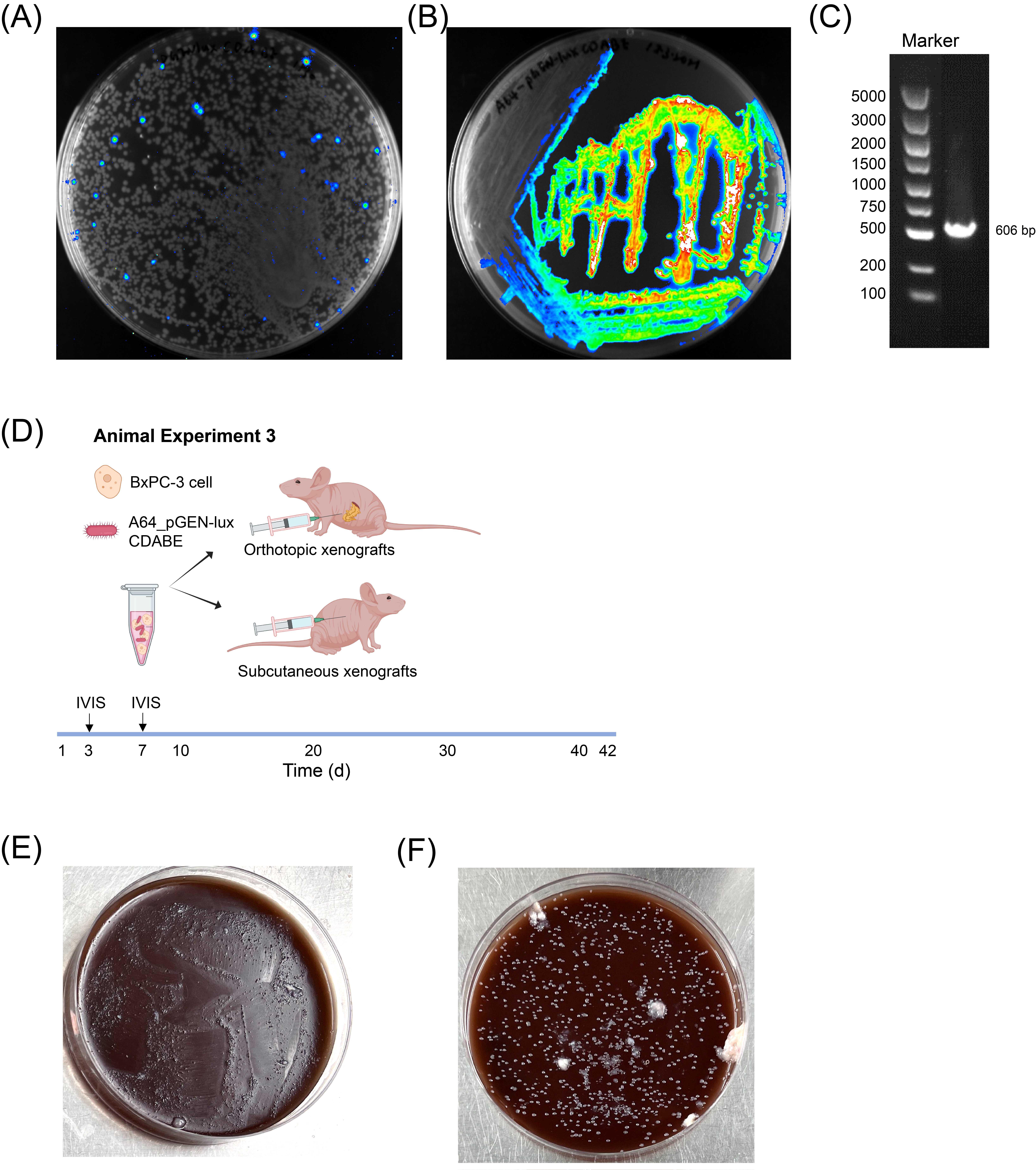


**Figure S12 Detection of intratumoral *E. hormaechei* proliferation and the effect of cefepime on tumor growth.** (A) A64_pGEN_luxCDABE colony was detected. (B) A64_pGENluxCDABE colony was passaged. (C) The right colony was verified by PCR amplification. (D) Schematic diagram of the Animal Experiment 3. (E) No bacteria were isolated from the tumor at the end point (*n* = 4). (F) Bacteria were isolated from the tumor at the end point (*n* = 4).


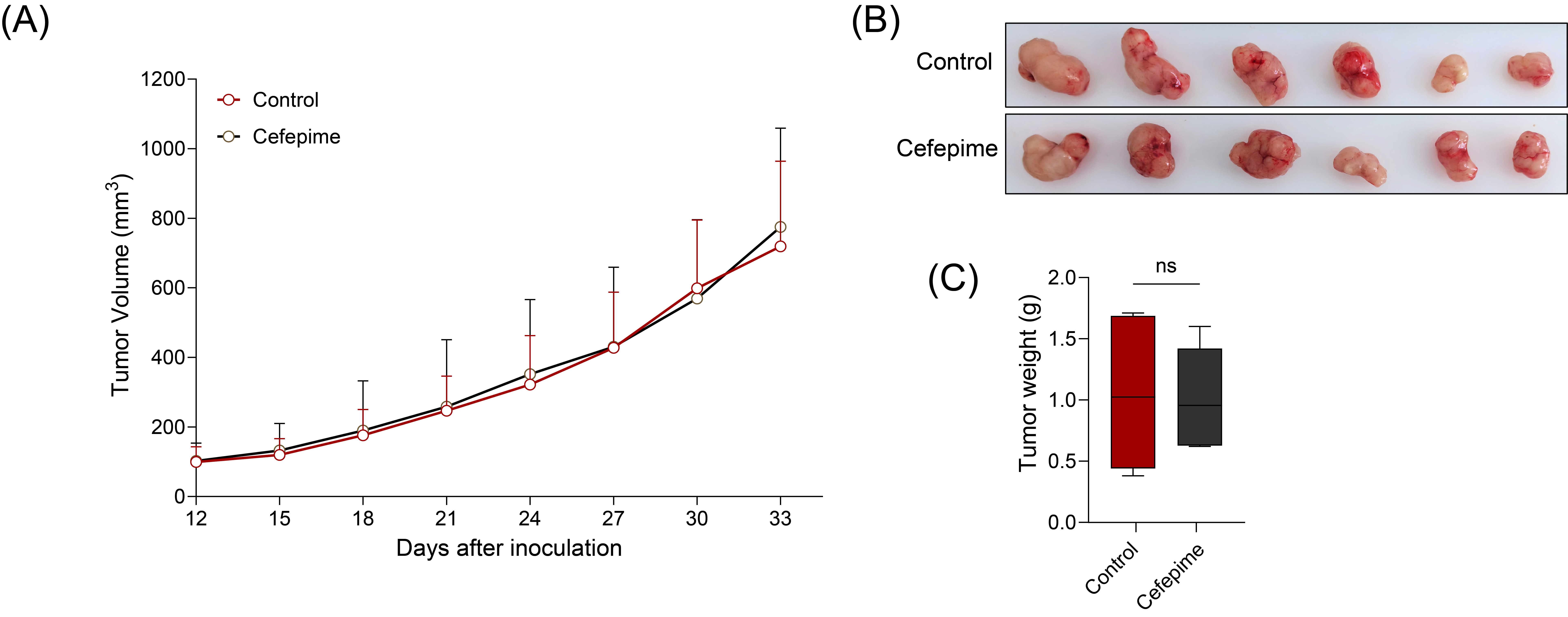


**Figure S13** **Antibiotics cannot inhibit or promote the growth of tumors**. (A) Tumor volume of mice treated with or without Cefepime (n = 6). (B) The morphology of the tumor at the endpoint (n = 6). (C) Tumor weight (n = 6).


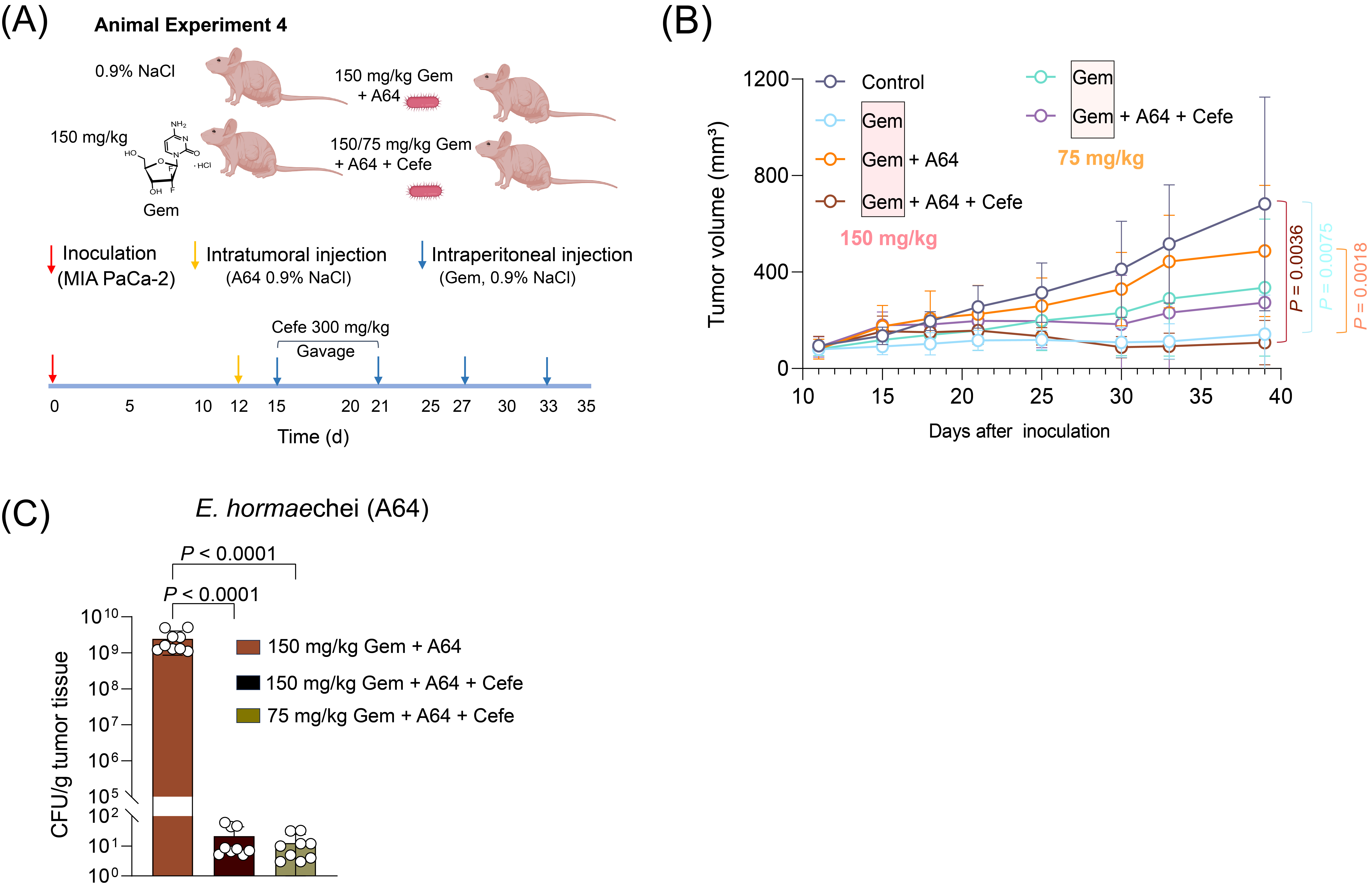


**Figure S14** **Antibiotic intervention eradicates intratumoral bacteria and restores gemcitabine efficacy *in vivo*.** (A) Schematic diagram of Animal Experiment 4 (n = 6). (B) Tumor volume was recorded at different time points (n = 6). (C) Bacteria within the tumor were quantified using three samples from each group (n = 3). *P* value was calculated by one-way ANOVA.





**Figure S15** **Immunohistochemistry demonstrates the widespread presence of *Enterobacter* spp. in various clinical solid tumor types.** (A) breast cancer, (B) ovarian cancer, (C) lung cancer, (D) esophagus cancer, and (E) melanoma. Technical repetitions (*n* = 5). (F) Quantitative analysis of IHC staining using IPP6.0.

**REFERENCES**

1. Vita, Alberto, Adolfo Amici, Tiziana Cacciamani, Marina Lanciotti and Giulio Magni. 1985. "Cytidine deaminase from *Escherichia coli* B. Purification and enzymatic and molecular properties." *Biochemistry* 24: 6020-4. https://doi.org/10.1021/bi00342a049
2. Li, Meixia, Yeqing Wang, Ciliang Guo, Sheng Wang, Liangzhen Zheng, Yifan Bu and Kan Ding. 2023. "The claim of primacy of human gut Bacteroides ovatus in dietary cellobiose degradation." *Gut Microbes* 15: 2227434. [https://doi.org/10.1080/19490976.2023.2227434](https://doi.org/10.1080/19490976.2023.22-27434)
3. Lyubimova, Anna, Shalev Itzkovitz, Jan Philipp Junker, Zi Peng Fan, Xuebing Wu and Alexander van Oudenaarden. 2013. "Single-molecule mRNA detection and counting in mammalian tissue." *Nature Protocol* 8: 1743–1758. https://doi.org/10.1038/nprot.2013.109
